# Does sauna bathing protect against dementia?

**DOI:** 10.1016/j.pmedr.2020.101221

**Published:** 2020-10-02

**Authors:** Paul Knekt, Ritva Järvinen, Harri Rissanen, Markku Heliövaara, Arpo Aromaa

**Affiliations:** a Finnish Institute for Health and Welfare, Helsinki, Finland; b University of Eastern, Finland

**Keywords:** Cohort study, Dementia, Passive body heating, Protection, Sauna

## Abstract

Repeated heat exposure like sauna bathing is suggested to beneficially affect against dementia development. The epidemiological evidence is, however, scarce. Therefore, we studied the association between heat exposure during sauna bathing (i.e., the frequency of sauna bathing, frequency of heat sessions, length of stay in heat, sauna temperature) and the subsequent risk of dementia. A prospective cohort study was conducted based on 13,994 men and women aged 30–69 and free from dementia diagnosis from the Finnish Mobile Clinic Follow-up Survey. During a follow-up of 39 years, a total of 1805 dementia patients were diagnosed. The sauna bathing data was gathered from a questionnaire. Analyses based on the Cox model included the sauna bathing variables and the potential confounding factors. Sauna bathing frequency was related to a reduced risk of dementia after adjustment for the potential sociodemographic, lifestyle, and metabolic risk factors of dementia considered. The hazard ratio of dementia between individuals sauna bathing 9–12 times per month in comparison with those not sauna bathing or sauna bathing less than four times per month was 0.47 (95% CI = 0.25–0.88) during the first 20 years of follow-up and 0.81 (95% CI = 0.69–0.97) during the whole follow-up. The results are in line with the hypothesis that sauna bathing provides protection against dementia. Further studies are required to verify the suggested benefits of sauna bathing.

## Highlights

• Frequent sauna bathing predicted decreased risk of dementia in a cohort from Finland.
• Result was independent of several dementia risk factors, and was not modified by sex.
• Findings support suggested benefits of sauna and passive body heating in the brain.

## 1 Introduction

The potential benefits of passive body heating in the brain have aroused interest recently (Coombs and Tremblay, 2019, Hunt et al., 2020). In Finland sauna bathing is an old tradition, and it represents one type/method of passive body heating.

In the Finnish sauna the individual is, in general, exposed to a dry heat of 70–100 °C for periods mostly varying between 5 and 20 min. The humidity of the sauna can be temporarily increased by casting water on the stones of the sauna heater standing in the corner of the sauna room. Heat sessions are repeated one to three times with cooling breaks of a few minutes outdoors, under a shower or swimming (Keast and Adamo, 2000, Laukkanen et al., 2018a). Saunas have always been considered a space for relaxation besides being a place to bath. The physiological effects of sauna bathing have been investigated since the fifties and some beneficial health effects of taking a sauna have been reported, but the quality of experiments has been variable and there has been a shortage of studies on the long-term effects of taking a sauna (Keast and Adamo, 2000, Hannuksela and Ellahham, 2001, Hussain and Cohen, 2018, Kukkonen-Harjula and Kauppinen, 2006). Recent findings of prospective studies from Finland suggest that frequent sauna bathing may reduce the risk of cardiovascular and other chronic diseases, including dementia (Laukkanen et al., 2018a). Among middle-aged male Finns, a moderate and high frequency of sauna bathing was associated with a lowered dementia risk during follow-up of twenty years (Laukkanen et al., 2017).

The present study was taken to estimate the strength of association between heat exposure during sauna bathing and the incidence of dementia in another population including men and women from Finland.

## 2 Methods

During the period 1973–1976, the Finnish Mobile Clinic Follow-up Survey (FMCF) carried out multiphasic health examinations in 12 municipalities from different parts of Finland (Reunanen et al., 1983). A total of 19,518 men and women aged 19 or over participated in the study. The participation rate was 79%. After inclusion of those aged 30–69 and free from a dementia diagnosis, the sample consisted of 13,994 individuals.

All participants completed a pre-mailed questionnaire that was checked at the baseline examination by trained nurses. The questionnaire yielded information about sauna bathing regarding the frequency of sauna baths per month and generally the number of heat sessions during one sauna bath, the length of one straight heat session, the temperature of the sauna, and the use of alcoholic beverages (i.e. how often, which beverages, and how much) in connection with the bathing. The questionnaire also provided information on demographic variables (education and marital status), lifestyle (leisure time physical activity, smoking, and total alcohol consumption), medication, chronic diseases (disease of the nervous system, atherosclerotic heart disease or cerebral stroke, diabetes, hypertension, and psychiatric disorders), and perceived health.

At the health examination, weight and height were measured, and body mass index (BMI, kg/m^2^) was calculated. Casual blood pressure was measured by a mercury manometer. Fasting blood samples were taken and stored at −20 °C for determination of serum total cholesterol and triglycerides and plasma fasting glucose (Reunanen et al., 1983).

A prospective cohort study design was chosen. The subsequent dementia diagnosis used as the outcome variable was ascertained through linkage with nationwide health registers. Dementia events (international classification of diseases [ICD]; revision 8, code 290; revision 9, codes 3310 and 4378A; revision 10, codes F00, F01, F02, F03, G30) leading to hospitalization were obtained from the Care Register for Health Care maintained by the Finnish Institute for Health and Welfare (Heliovaara et al., 1984, Solomon et al., 2014). Information on mortality with dementia as an underlying or contributory cause of death was based on death certificates obtained from Statistics Finland (Reunanen et al., 1983). Information on prescribed anti-dementia drugs and reimbursed Alzheimer’s medication was obtained from the Social Insurance Institution’s registries. The follow-up time was defined as the number of days from the baseline examination to the date of dementia occurrence, death, or end of follow-up, whichever came first. During a 39-year follow-up from 1973 to 2011, 1805 dementia cases were identified. Of these 1343 dementia patients were included in the Care Register for Health Care, 834 in death certificates from Statistics Finland, and 858 were Social Insurance Institution’s registries on medication. A total of 784 only came from one of the registers. The registers do not include information from health centers or private outpatient clinics.

The FMCF (Reunanen et al., 1983) precedes the current legislation on ethics in medical research. All participants were fully informed about the study, they participated in the study voluntarily, and the use of the information for medical research was explained to them. Agreeing to participate in the baseline health examination was taken to indicate informed consent. The participants were free to unconditionally withdraw their consent at any time, in which case their data were deleted. The study protocol and the practice of the subject’s voluntary participation indicating informed consent were approved by the Institutional Review Board (IRB 00007085) of the Finnish Institute for Health and Welfare.

## 3 Statistical methods

The description of the distribution of sauna bathing by potential confounding factors was carried out using linear regression with the continuous frequency of sauna bathing as the dependent variable and the respective potential confounding factor as the independent variable. The Cox model was used to assess the hazard ratio (HR) and 95% confidence interval (95% CI) of dementia between categories of indicators for sauna bathing and potential confounding factors at baseline. Model 1 included the sauna variable considered, age, and gender. Model 2 further included variables satisfying criteria for confounding (Rothman et al., 2008) (education, marital status, region type, leisure-time physical activity, smoking, alcohol consumption, body mass index, hypertension, plasma fasting glucose, serum triglycerides, and total serum cholesterol). Also models adjusting for chronic diseases (diseases of the nervous system, atherosclerotic heart disease or cerebral stroke, diabetes, hypertension, and psychiatric disorders) were carried out (data not shown due to no effects). The potential effect modification of age, gender, education, chronic disease, and the different sauna bathing indicators on the association between sauna bathing and dementia incidence was studied by including first order interaction terms in the second model. The proportional hazards assumption was studied by carrying out analyses for different lengths of follow-up (i.e . for 20, 25, 30, 35, and 39 years) (data shown only for 20 and 39 years). The analyses were carried out using SAS software version 9.3 (SAS Institute Inc., Cary, North Carolina).

## 4 Results

The majority of individuals in the present study had basic education (Table 1). The participants also had a high prevalence of smoking and low prevalence of regular leisure time physical activity. They also on average had elevated blood pressure and a high risk of atherosclerotic heart disease or cerebral stroke.

**Table 1:** Baseline characteristic of participants^a^ in the Finnish Mobile Clinic Follow-up Survey.

Variable, unit	Mean (SD)
**Demographic variables**	
Male gender, %	51.2
Age, years	47,9 (11.0)
Basic education, %	70.7
Married, %	77.8
**Lifestyle**	
Regular leisure time physical activity, %	11.9
Current smoker, %	26.8
Alcohol consumption, g/week	42.3 (79.0)
**Metabolic health**	
Body mass index, kg/m^2^	25.9 (4.1)
Systolic blood pressure, mmHg	146 (24.0)
Plasma fasting glucose, mmol/l	5.52 (1.13)
Serum triglycerides, mmol/l	1.45 (0.95)
Serum total cholesterol, mmol/l	6.99 (1.37)
**Chronic diseases**	
Disease of nervous system, %	7.68
Atherosclerotic heart disease or cerebral stroke, %	17.2
Diabetes, %	1.94
Psychiatric disorder, %	3.48

a Includes 30–69 years old participants (N = 13,994).

The strength of association for several demographic, lifestyle, and metabolic variables with dementia occurrence satisfied criteria for confounding (Table 2).

**Table 2:** Hazard ratio (HR)^a^ of dementia between categories of potential risk factors in the Finnish Mobile Clinic Follow-up Survey.

Variable	Category	Number of dementia	Number at risk	HR	95%CI
**Demographic variables**					
Sex	Male	707	7023	1	
	Female	1064	6740	0.87	0.77–0.98
Age, years	30–39	118	3830	1	
	40–49	536	3938	5.84	4.77–7.15
	50–59	682	3400	16.6	13.5–20.4
	60–69	435	2595	55.3	44.0–69.5
Education	Basic	1358	9703	1	
	Intermediate	313	3187	0.82	0.72–0.93
	Higher	100	873	0.83	0.67–1.02
Marital status	Unmarried	180	1605	1	
	Married	1359	10,720	0.96	0.82–1.13
	Widow	177	959	0.87	0.70–1.08
	Divorced	55	479	0.85	0.63–1.16
Region type	Urban	758	5633	1	
	Rural	639	4971	0.87	0.78–0.97
	Industrial	374	3159	0.85	0.75–0.97
**Lifestyle**					
Leisure time physical activity	Inactive	459	3527	1	
	Occasionally	1125	8691	0.99	0.89–1.11
	Regularly	187	1645	0.98	0.82–1.17
Smoking	Never	1140	7448	1	
	Former	312	2624	0.97	0.83–1.12
	Current	319	3691	1.25	1.08–1.44
Alcohol	None	1016	6189	1	
consumption^b^	Moderate	698	6876	0.83	0.74–0.93
	Heavy	57	698	1.00	0.76–1.33
**Metabolic health**					
Body mass index	<25	701	6287	1	
(kg/m^2^)	25–29.9	769	5513	0.97	0.87–1.08
	≥30	301	1963	1.08	0.94–1.25
Blood pressure^c^	Normal	555	5253	1	
	Elevated	1216	8510	1.08	0.97–1.21
Plasma fast. glucose^d^	<5.6	1093	8905	1	
(mmol/l)	≥5.6	678	4858	1.15	1.05–1.28
Serum triglycerides^d^	<1.7	1318	10,334	1	
(mmol/l)	≥1.7	453	3429	1.12	1.00–1.25
Serum cholesterol	<6	333	3305	1	
(mmol/l)	≥6	1438	10,458	0.96	0.85–1.08

Diabetes Federation (Alberti et al., 2006).

a The model included all variables in the table and the number at risk (N = 13763) included all individuals with no missing data in any of the variables in the model.

b Total alcohol consumption: None = 0; Moderate = 1–199 g/week for men and 1–99 g/week for women; Heavy ≥ 200 g/week for men and ≥ 100 g/week for women.

c Blood pressure was considered elevated if systolic pressure was ≥ 140 or diastolic pressure ≥ 90 or antihypertensive medication was used.

d The cut-off was based on the definition of metabolic syndrome, according to the International

Virtually all the participants (99.0%) practiced sauna bathing on average 6.03 (SD = 2.73) times per month. The mean number of sauna baths differed between categories of the potential confounding factors considered (Table 3). Generally, one to two heat sessions were taken, the straight stay in heat per one heat session lasted under 15 min, and the temperature of the sauna was below 100 °C (Table 4). About one third of the participants reported the use of beer or other alcoholic beverages in connection with sauna bathing.

**Table 3:** Frequency of sauna bathing in categories of demographic, lifestyle, and metabolic factors in the Finnish Mobile Clinic Follow-up Survey.

Variable	Category	Sauna bathing frequency per month^a^		
		Number	Mean	SD
**Demographic variables**				
Sex	Men	7004	6.11	2.82
	Women	6689	5.97	2.64
				
Age group, years	30–39	3813	6.48	2.90
	40–49	3922	6.38	2.70
	50–59	3380	5.80	2.71
	60–69	2578	5.18	2.29
				
Region type	Rural	5605	5.77	2.71
	Urban	4950	6.34	2.70
	Industrial	3138	6.04	2.81
				
Education	Basic	9651	6.10	2.74
	Intermediate	3172	5.98	2.72
	Higher	870	5.55	2.80
				
Marital status	Unmarried	1593	5.23	2.77
	Married	10,669	6.22	2.70
	Widow	954	5.76	2.58
	Divorced	477	5.20	2.90
				
**Lifestyle**				
Leisure time physical activity	Inactive	3516	5.80	2.80
	Occasionally	8539	6.19	2.70
	Regularly	1638	6.59	2.72
				
Smoking	Never	7399	6.08	2.63
	Former	2611	6.09	2.81
	Current	3683	5.93	2.88
				
Alcohol consumption^b^	None	6144	5.81	2.60
	Moderate	6855	6.19	2.78
	Heavy	694	6.59	3.13
**Metabolic health**				
Body mass index	< 25	6256	5.87	2.71
(kg/m^2^)	25–29.9	5482	6.17	2.80
	≥ 30	1955	6.22	2.64
				
Blood pressure^c^	Normal	5228	6.04	2.75
	Elevated	8465	5.04	2.72
				
Plasma fasting glucose^d^	< 5.6	8861	6.10	2.73
(mmol/l)	≥ 5.6	4832	5.81	2.74
				
Serum triglycerides^d^	< 1.7	10,284	6.11	2.75
(mmol/l)	≥ 1.7	3409	5.84	2.69
				
Serum total cholesterol	< 6	3283	5.88	2.66
(mmol/l)	≥6	10,410	6.09	2.76

a All variables were included in the same linear model and the number at risk (N = 13693) included all individuals with no missing data in any of the variables in the model. There were differences between the categories in all variables.

b Total alcohol consumption: None = 0; Moderate = 1–199 g/week for men and 1–99 g/week for women; Heavy ≥ 200 g/week for men and ≥ 100 g/week for women.

c Blood pressure was considered elevated if systolic pressure was ≥ 140 or diastolic pressure ≥ 90 or antihypertensive medication was used.

d The cut-off was based on the definition of metabolic syndrome, according to the International Diabetes Federation (Alberti et al., 2006).

**Table 4:** Hazard ratio (HR) of dementia in categories of indicators of sauna bathing in the Finnish Mobile Clinic Follow-up Survey.

			Sex- and age-adjusted^a^	Full model^b^
Indicator	n	N	HR (95% CI)	HR 95%CI
**Frequency of sauna baths/month**				
≤ 0–4 times	660	4724	1	1
5–8 times	926	7028	0.97 (0.87–1.07)	1.01 (0.90–1.11)
9–12 times	178	1896	0.76 (0.64–0.90)	0.81 (0.69–0.97)
13–30 times	29	271	0.90 (0.62–1.30)	0.91 (0.62–1.33)
**Number of heat sessions/sauna bath**				
Once	1108	7949	1	1
2 times	584	5123	0.96 (0.87–1.06)	0.98 (0.88–1.09)
3 times or more	88	763	0.92 (0.74–1.15)	0.95 (0.76–1.18)
**Length of one heat session straight**				
< 5 min.	401	2686	1	1
5–14 min.	1206	9876	0.86 (0.77–0.97)	0.90 (0.80–1.01)
≥ 15 min.	154	1161	0.86 (0.71–1.04)	0.87 (0.72–1.05)
**Temperature of sauna**				
< 80 °C	718	5108	1	1
80–99 °C	963	8019	0.93 (0.85–1.03)	0.94 (0.85–1.04)
> 100 °C	92	653	1.11 (0.89–1.38)	1.14 (0.92–1.43)

a The number at risk depend on the number of individuals without missing data in respective bathing variable.

b Full model includes sex, age, education, marital status, region type, leisure time physical activity, smoking, alcohol consumption, body mass index, blood pressure, plasma fasting glucose, serum triglycerides, and serum total cholesterol.

Sauna bathing was related to a reduced risk of dementia after adjustment for potential sociodemographic, lifestyle, and metabolic risk factors of dementia (Table 4). The hazard ratio (HR) among those bathing 9–12 times per month was 0.81 (95% CI = 0.69–0.97) compared to those bathing 0–4 times per month. The HR of participants bathing 13–30 times per month did not differ from those with a monthly bathing frequency of 0–4 times. A straight stay in heat for 5–14 min per heat session vs. < 5 min was suggestively related to a reduced risk. The most favorable sauna temperature for dementia protection was 80–99 °C.

A study on the strength of association of sauna variables with subsequent dementia occurrence during different follow-up periods showed the strongest association for the frequency of sauna baths per month and temperature of sauna during the first 20 years of follow-up. The HR of dementia for individuals bathing 9–12 times per month in comparison with those bathing 0–4 times was 0.47 (95% CI = 0.25–0.88). For high sauna temperatures of 100 °C or more, an elevated risk was revealed during the first 20 years (HR = 2.04, 95% CI = 1.32–3.15) in comparison with those with a temperature under 80 °C.

Gender, age, education, perceived health, or chronic diseases did not notably modify the association between frequency of sauna bathing and dementia occurrence (data not shown).

## 5 Discussion

The results of our study suggest that frequent sauna bathing may reduce the risk of dementia in men and women. This finding is in line with the results of a previous study on middle-aged men from Finland (Laukkanen et al., 2017).

Of the different features of sauna bathing habits considered, the frequency of sauna bathing was the most important predictor of dementia. During the first 20 years of follow-up, the dementia risk of those reporting 9–12 sauna baths per month (i.e., approximately three per week) was less than a half of the risk of those who had sauna baths only 0–4 times per month. The reduction in the dementia risk was attenuated during the follow-up, but the decrease of the risk was still evident after nearly 40 years. Accordingly, a sauna bathing frequency of three times per week may be associated with a reduced risk of dementia.

Appropriate heat exposure is apparently needed to attain the suggested benefits of sauna bathing. However, sauna heat which is too high may not be good for the brain. The dementia risk of those bathing in sauna temperatures higher than 100 °C doubled compared to those bathing at temperatures lower than 80 °C during the first twenty years of follow-up.

Passive body heating elicits several physiological, metabolic, and cellular changes which may affect brain function. The importance of these mechanisms in the dementia development however remains to be ascertained (Hunt et al., 2020).

The elevation of body temperature either due to increased metabolism caused by physical exercise or due to passive body heating induces the appearance of heat shock (Noble et al., 2008, Faulkner et al., 2017, Brunt et al., 2018, Oehler et al., 2001). Heat shock proteins are important regulators in normal cell functions and have an essential role in guarding and controlling protein formation (Schlesinger, 1990, Stetler et al., 2010). Because disturbances of protein construction and folding are central to the development of neurological diseases, heat shock proteins may be important in maintaining protein homeostasis in the brain (Kampinga and Bergink, 2016, Hunt et al., 2020).

An adequate blood supply and vascular factors are important not only in vascular dementia but also in Alzheimer’s disease (Kapasi and Schneider, 2016, Shabir et al., 2018). Stress induced experimentally by passive body heating in a warm water bath has been shown to strengthen the cardiovascular function, improving the endothelial function, arterial stiffness and blood pressure (Brunt et al., 2016). Exposure to Finnish sauna heat is suggested to cause similarly beneficial effects on the cardiovascular function (Laukkanen et al., 2018c, Lee et al., 2018). In a recent cross-sectional study greater carotid intima-media thickness reflecting atherosclerotic changes in the veins was associated with lower cerebral blood flow (Cermakova et al., 2020). Prospectively, frequent sauna bathing has been associated with a reduced risk of hypertension (Zaccardi et al., 2017) and cardiovascular diseases (Laukkanen et al., 2018b).

Inflammatory processes are suggested to be important in neurodegenerative diseases (Chen et al., 2016). In cross-sectional and longitudinal studies sauna bathing was associated with lower levels of inflammatory serum markers implicating reduced inflammation among those bathing frequently (Laukkanen and Laukkanen, 2017, Kunutsor et al., 2018). It is possible that some of the effects of sauna in the brain are conveyed via reduced inflammation.

Sauna bathing and other passive body heating are reported to affect several endocrine variables, which may also contribute to suggested benefits in the brain. The effects of body passive heating on hormones, however, are greatly variable and depend on individual features of the bathers and circumstances of body heating, and adaptation to sauna affects the responses (Huhtaniemi and Laukkanen, 2020). An increase of beta-endorphin level frequently reported during sauna bathing apparently contributes to the feeling of well-being after sauna (Laukkanen et al., 2018a). Plasma stress hormones, e. g. cortisol are increased as a respond to heat stress, but the effect depends on test conditions (Huhtaniemi and Laukkanen, 2020). Sauna bathing with a long duration may be a wearing load for the body (Rissanen et al., 2020).

Problems with sleeping and lack of sleep are often associated with Alzheimer’s disease and dementia (Shi et al., 2018). An increased risk of dementia was associated with frequent sleep disturbances in a study of middle-aged men from Finland during a 20-year follow-up (Luojus et al., 2017). Findings from experiments which used warm water baths for passive body heating suggest that an increase in the body core temperature beneficially affects sleep, depending on the increase in body core temperature and the proximity to sleep (Horne and Reid, 1985, Bunnell et al., 1988, Liao, 2002). Potentially sauna bathing affects sleeping similarly. In a survey of middle-aged urban dwellers in Finland, sauna bathing was mentioned among the factors that were perceived to promote sleep (Urponen et al., 1988).

The possible mechanism may also be related to enhanced cognitive reserve (Xu et al., 2020), increased social contacts (Kuiper et al., 2015), and stress reduction (Escher et al., 2019), all dementia related factors not measured in this study. Apparently, these factors may also be related to sauna bathing. Relaxation and beneficial effects on mood and wellbeing habitually associated with sauna bathing and other passive body heating may contribute to the suggested benefits of sauna on the brain, though research data on this topic is limited (Hussain and Cohen, 2018, Laukkanen et al., 2018a). Stress reduction, enjoyment, and social contacts were the most often named motivations for sauna bathing by the respondents of an online questionnaire (Hussain et al., 2019). As such, mild to moderate heat stress may improve cognition, whereas severe heat stress is suggested to impair cognitive performance (Schmit et al., 2017).

Although a major part of research on the effects of sauna bathing is in favor of the benefits of the habit, it cannot be excluded that strong passive body heating could have harmful effects in the brain. Severe heat stress may result in a reduction of the cerebral blood flow and may increase blood–brain barrier permeability (Nelson et al., 2011, Bain et al., 2015). It is thus possible that during bathing at very high sauna temperatures, the body core temperature raises high enough to harmfully affect cerebral blood flow. The elderly and other groups with compromised thermoregulatory control are especially vulnerable during severe passive heat stress (Bain et al., 2015). Among young people the effects of moderate and high thermal stress on the brain may be counterbalanced by cardiovascular, cerebral and metabolic alterations (Bain et al., 2020, Gibbons et al., 2020).

Our study has several strengths: the large population based sample including men and women, the longitudinal cohort study design with lengthy follow-up of the occurrence of dementia, the comprehensive set of variables describing sauna bathing, and the availability of several potential confounding factors. Due to continuous follow-up through national registries there were no drop-outs.

There are, however, also limitations. The small number of dementia cases in some subgroups of the study population may have hidden part of the associations. Only a single measurement of sauna bathing was available at baseline, which fails to take into account possible intra-individual changes in the habit during the long follow-up, thus possibly confounding the strength of associations. The risk factors of dementia are not well known and, therefore, despite comprehensive adjustments for potential confounders and consideration of potential effect-modifying factors, residual confounding may still remain. Since the dementia diagnoses were based on register data, the incidence obviously has been underestimated and, consequently, the estimates of the strength of association between sauna bathing and dementia incidence may be conservative. Despite, diagnoses of dementia covered by the registers have been shown to be accurate enough for cohort studies like the current one (Solomon et al., 2014). The prevalent dementia cases at baseline could not fully be eliminated. To minimize this effect, we excluded all individuals who were 70 or above from the study. Reliable separation of different types of dementia was not possible based on the register data available. Alzheimer’s disease and vascular dementia often share some features of pathology and most cases of dementia are, in fact, mixed. As our study was carried out in Finland where sauna bathing belongs to the weekly routines of nearly all inhabitants from childhood to old age (Markkola et al., 1989, Strandberg et al., 2018), the findings may only be relevant in these circumstances. Due to the very low number of participants who did not use sauna at all, the eventual consequences of not sauna bathing could not be studied among this population.

## 6 Conclusion

The findings of this population-based study, including men and women from Finland, are in line with the hypothesis that sauna bathing provides protection against the development of dementia. However, because of the lack of exact hypotheses on the etiology of the association, the lack of repeated measurements of sauna bathing during the follow-up, the possible residual confounding, and the complexity of the dementia diagnosis available, no firm conclusions can be made. Further results from large-scale cohort studies with repeated measurement on sauna exposure are needed.

## 7 Ethics committee approval

All procedures performed were in accordance with the ethical standards of the institutional research committee and with the 1964 Declaration of Helsinki and its later amendments or comparable ethical standards.

## CRediT authorship contribution statement

**Paul Knekt:** Conceptualization, Methodology, Formal analysis, Investigation, Writing - original draft. **Ritva Järvinen:** Writing - original draft. **Harri Rissanen:** Methodology, Resources, Data curation, Writing - review & editing. **Markku Heliövaara:** Investigation, Writing - review & editing. **Arpo Aromaa:** Conceptualization, Investigation, Writing - review & editing.

## Declaration of Competing Interest

The authors declare that they have no known competing financial interests or personal relationships that could have appeared to influence the work reported in this paper.
